# Metabolomic profiles differ among unique genotypes of a threatened Caribbean coral

**DOI:** 10.1038/s41598-019-42434-0

**Published:** 2019-04-15

**Authors:** Kathryn E. Lohr, Ram B. Khattri, Joy Guingab-Cagmat, Emma F. Camp, Matthew E. Merritt, Timothy J. Garrett, Joshua T. Patterson

**Affiliations:** 1 Program in Fisheries and Aquatic Sciences, School of Forest Resources and Conservation, University of Florida/IFAS, Gainesville, FL USA; 2 Department of Biochemistry and Molecular Biology, College of Medicine, University of Florida, Gainesville, FL USA; 3 Department of Pathology, Immunology, and Laboratory Medicine, College of Medicine, University of Florida, Gainesville, FL USA; 4 Climate Change Cluster, University of Technology Sydney, Ultimo, NSW Australia; 5 Center for Conservation, The Florida Aquarium, Apollo Beach, FL USA

## Abstract

Global threats to reefs require urgent efforts to resolve coral attributes that affect survival in a changing environment. Genetically different individuals of the same coral species are known to exhibit different responses to the same environmental conditions. New information on coral physiology, particularly as it relates to genotype, could aid in unraveling mechanisms that facilitate coral survival in the face of stressors. Metabolomic profiling detects a large subset of metabolites in an organism, and, when linked to metabolic pathways, can provide a snapshot of an organism’s physiological state. Identifying metabolites associated with desirable, genotype-specific traits could improve coral selection for restoration and other interventions. A key step toward this goal is determining whether intraspecific variation in coral metabolite profiles can be detected for species of interest, however little information exists to illustrate such differences. To address this gap, we applied untargeted ^1^H-NMR and LC-MS metabolomic profiling to three genotypes of the threatened coral *Acropora cervicornis*. Both methods revealed distinct metabolite “fingerprints” for each genotype examined. A number of metabolites driving separation among genotypes were identified or putatively annotated. Pathway analysis suggested differences in protein synthesis among genotypes. For the first time, these data illustrate intraspecific variation in metabolomic profiles for corals in a common garden. Our results contribute to the growing body of work on coral metabolomics and suggest future work could identify specific links between phenotype and metabolite profile in corals.

## Introduction

The global decline of coral reefs demands novel tools to understand these ecosystems and their ability to persist under present and future environmental conditions^1–3^. Ongoing threats to the survival of coral reefs include increasing global sea temperature^4,5^, prevalence of diseases^6^, and frequency and intensity of tropical storms^7,8^. Importantly, there is growing evidence to indicate intraspecific differences in the ability of corals to withstand these stressors^9–11^. Additional data on the physiological mechanisms underlying within-species variability in coral performance could aid in understanding how corals respond to such threats, and can inform conservation strategies such as selective breeding, transplantation, assisted gene flow, and coral restoration^2,3^. Metabolomic profiling is a technique that provides a snapshot of an organism’s physiological state at a given point in time^12^. Changes in metabolomic profiles can be a result of altered gene expression^13,14^, but may also be due to post-transcriptional processes^12^. Although metabolomics approaches have only recently been applied to the reef-building coral holobiont^13,15–17^, they have clear applications for studies of these organisms.

Physiological responses in corals can be particularly complex, given that corals are holobionts, comprised of the host organism, photosynthetic dinoflagellate symbionts, and an associated microbial community^18^. Each of these components is known to play a significant physiological role^18,19^, and therefore physiological studies that consider the complete holobiont are valuable. A recent study compared entire-holobiont metabolomic profiles among four coral species, indicating distinct profiles for each taxon^13^. Similarly, metabolomics has been used to explore shifts in the holobiont metabolome in response to contact with competitive algae^17^. The metabolomes of individual holobiont components have also been considered separately. For example, metabolomic profiling has been used to explore the role of dinoflagellate symbiont and microbial communities in cnidarian biology and physiology^20–22^. The cnidarian metabolome has also been shown to change in response to external stimuli, such as exposure to abiotic conditions consistent with predicted climate change^23,24^. Although metabolomic profiling has provided a range of new information on coral physiology, no study has yet illustrated intraspecific variation in the metabolome of any coral species. Given the strong linkages between genome and metabolome^12^ and our understanding that genetically distinct individuals can respond differently to the same environment^25,26^, it is reasonable to anticipate that genetically unique corals in a common garden will exhibit differing suites of metabolites.

The link between genotype and metabolome has been explored in a variety of organisms. For example, high intraspecific metabolomic variability was found for the model bacterium species *Myxococcus xanthus*^27^. Similarly, unique metabolomic profiles were found for genetically distinct populations of *Arabidopsis lyrata* ssp. *petraea*, and these differences were associated with the ability of each population to withstand low temperatures^28^. Intraspecific variation in metabolomic profiles was also found among barley genotypes, with implications for disease resistance^29^. Variation in metabolomic profiles among genotypes is prerequisite to screen for desirable traits like disease resistance using specific metabolite biomarkers^29^. Such strategies could also extend to corals, which have been shown to possess intraspecific variation in key traits that could affect fitness^9,10,25,26,30,31^. However, no study has yet demonstrated that metabolomic profiles differ among genetically distinct corals of the same species in a common garden, a key first step toward linking metabolome and phenotype in corals.

We employed proton-nuclear magnetic resonance spectroscopy (^1^H-NMR) and liquid chromatography-mass spectrometry (LC-MS) to identify and compare metabolomic profiles for three unique genotypes of the threatened staghorn coral *Acropora cervicornis* in a common garden coral nursery. While ^1^H-NMR is less sensitive than LC-MS, it is ideal for quantifying and identifying the structure of unknown compounds^32^. ^1^H-NMR is therefore useful for untargeted metabolomic studies^33^, particularly of organisms such as corals that lack extensive databases for compound identification. However, LC-MS is far more sensitive, and can therefore resolve a higher number of metabolites, particularly less abundant compounds like secondary metabolites^33^. Application of both ^1^H-NMR and LC-MS in tandem therefore improves coverage of the metabolome and enhances both metabolite resolution and identification. This exploratory work builds upon a previous study in which growth and thermotolerance were characterized among these *A*. *cervicornis* genotypes at the same nursery^25^. We hypothesized that each of the three genotypes tested would have unique metabolomic profiles. These data increase our basic knowledge of the coral metabolome and represent an important step toward linking genotype, phenotype, and metabolome in reef-building corals.

## Results

During sample collection (~2 hours), temperature at the depth of the nursery trees was 26.5–26.6 °C. Mean daily temperature ranged from 24.8–30.9 °C during the months preceding sample collection (i.e. Jul–Dec).

### ^1^H-NMR Profiling

**Figure 1:**
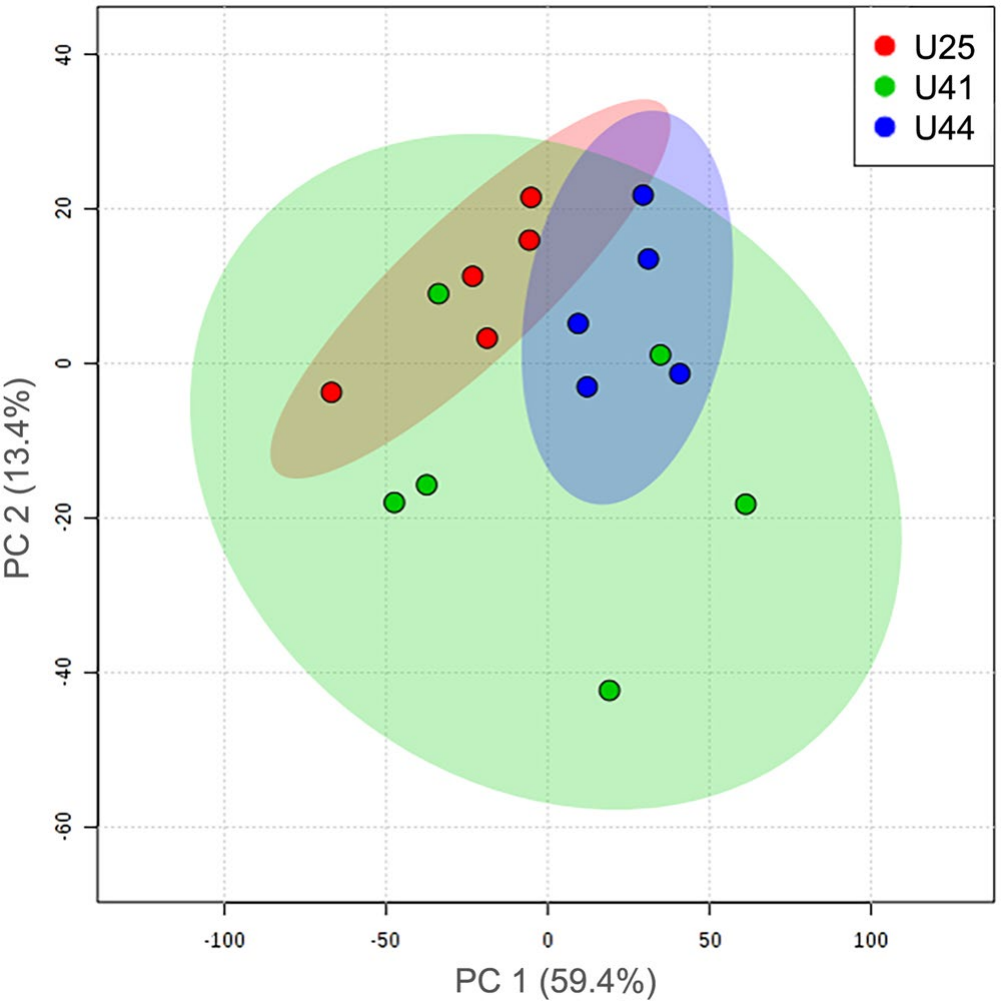
Principal component analysis model comparing ^1^H-NMR metabolomic profiles among three unique genotypes of *A*. *cervicornis*: U25 (red), U41 (green), and U44 (blue). The amount of variance explained is shown in parentheses on each axis.

**Figure 2:**
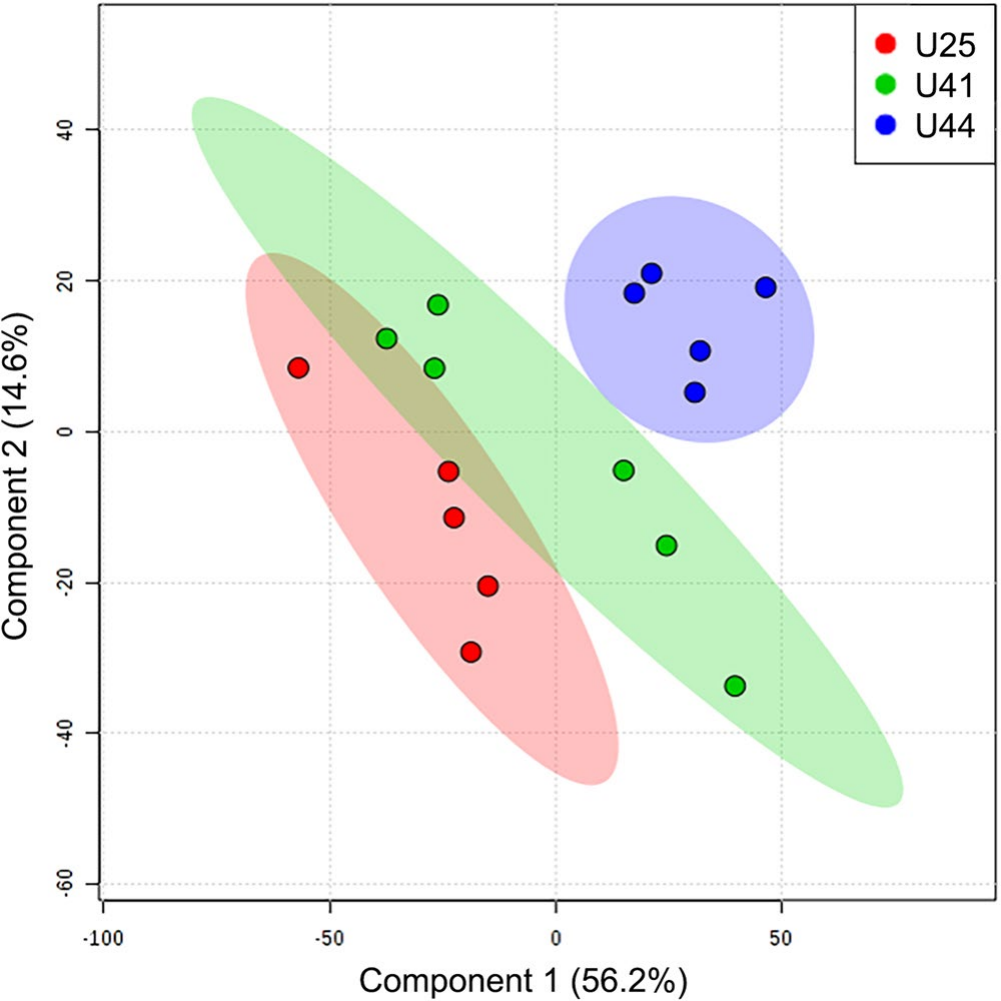
Partial least square discriminant analysis model comparing ^1^H-NMR metabolomic profiles among three unique genotypes of *A*. *cervicornis*: U25 (red), U41 (green), and U44 (blue). The amount of variance explained is shown in parentheses on each axis.

A False Discovery Rate (FDR) corrected analysis of variance (ANOVA) model found 59 chemical shifts, representing signals derived from coral metabolites that differed significantly among genotypes (*p* < 0.05). Principal component analysis (PCA) and partial least square discriminant analysis (PLS-DA) were used to assess metabolomic data. PCA is an unsupervised dimension reduction method that seeks to explain the maximum variation in a multivariate dataset without *a priori* information on sample groups^34^. PCA therefore provides an overview of variation in datasets, but principal components may not identify variables driving maximum separation among groups, as treatments/classes are not accounted for in the PCA algorithm^34^. In contrast, PLS-DA is a supervised method that seeks to maximize separation among known groups^34^ (i.e. genotypes in the present study). PLS-DA models maximum covariance between variables (i.e. metabolites) and treatment groups (i.e. genotypes) to best understand factors driving separation^34^. Thus, use of both PCA and PLS-DA provides a more comprehensive analysis of patterns in metabolomic profiles among genotypes. Combined, PCA components 1 and 2 explained 71.6% of the total variance among genotypes. PCA indicated relatively separate clustering for genotypes U25 and U44, however U41 had greater within-group variability compared to the other two genotypes, and overlapped with both (Fig. 1). The PLS-DA model was well-validated using a permutation test and had Q^2^ > 0.6. PLS-DA revealed separate clustering of all three genotypes, and components 1 and 2 explained 70.8% of the total variance among genotypes (Fig. 2). Genotype U41 had a greater spread across components 1 and 2 compared to the other two genotypes, again suggesting relatively greater within-group variability. Compounds driving separation in the PLS-DA model (with a VIP score > 2) are presented in Table 1. Compounds were identified and quantified from ^1^H-NMR spectra using Chenomx NMR Suite 8.2 (Chenomx, Inc.). A full list of compounds identified by ^1^H-NMR, including putative metabolite identifications, is provided as Supplementary Data.

**Table 1:** Chemical shifts driving separation of ^1^H-NMR metabolomic profiles among genotypes determined by partial least square discriminant analysis.

Spectra Bin	Metabolite	Metabolite Class	Peak pattern	U25	U41	U44
3.23	Trimethylamine N-oxide	Organic compound	s			
2.95	Unknown	Unknown	s			
2.99	Unknown	Aliphatic	s			
2.70	Unknown	Carbohydrate	m			
3.27	Unknown	Carbohydrate	m			
3.54	Unknown	Carbohydrate	t			
3.47	Unknown	Carbohydrate	s			
3.39	Unknown	Carbohydrate	d			
3.19	Choline	Aliphatic	s			
3.11	Malonate	Aliphatic	s			
3.15	N-Nitrosodimethylamine	Aliphatic	s			
2.75	Unknown	Aliphatic	s			
2.59	Methylamine	Aliphatic	s			
2.03	Homoserine	Aliphatic	m			
2.83	Unknown	Aliphatic	s			

For peak pattern, s = singlet, d = doublet, t = triplet, m = multiplet. Circle size illustrates the relative concentration of each metabolite compared to other genotypes (smallest circles = low, largest circles = high).

### LC-MS Global Metabolomics

**Figure 3:**
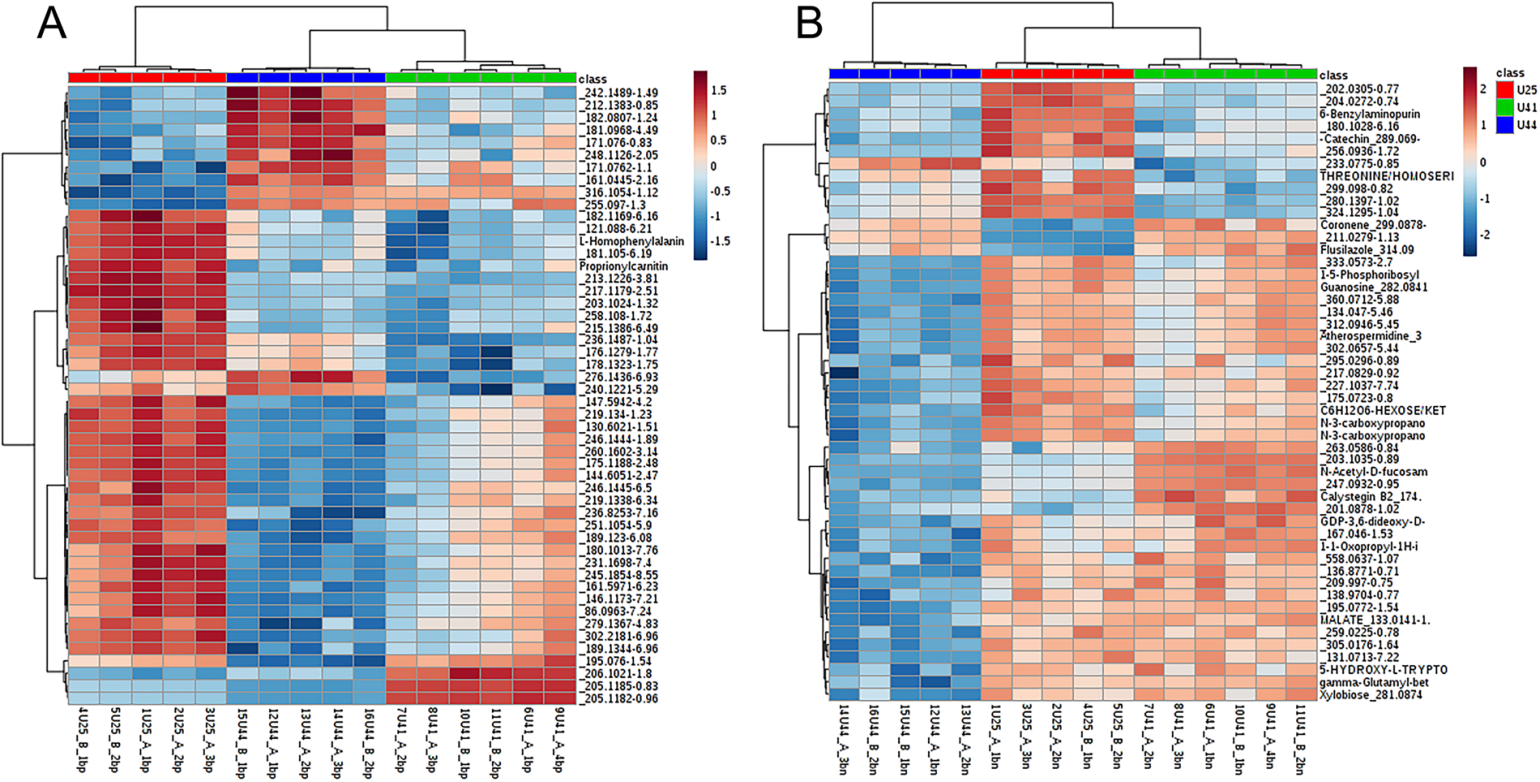
Heat map showing differences in concentrations among genotypes for 50 significant features identified via analysis of variance from LC-MS in the positive (left) and negative (right) ion modes. The scale bar represents normalized intensity of features. This figure illustrates a subset of metabolites that varied among genotypes, but does not depict the full list of features that varied significantly among genotypes. A full list of metabolites that varied among genotypes is provided as Supplementary Data.

**Figure 4:**
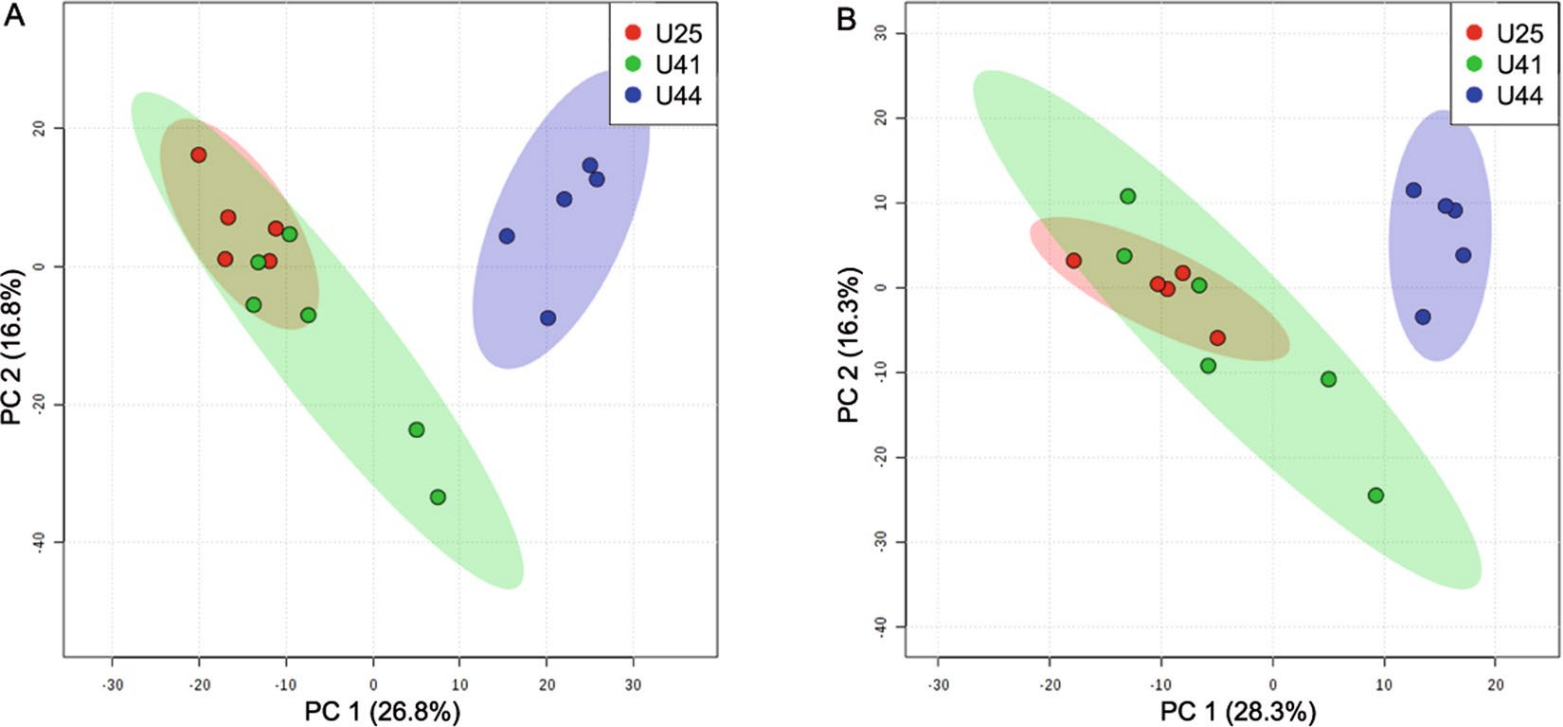
Principal component analysis model comparing LC-MS metabolomic profiles for the positive (left) and negative (right) mode among three unique genotypes of *A*. *cervicornis*: U25 (red), U41 (green), and U44 (blue). The amount of variance explained is shown in parentheses on each axis.

LC-MS detected a total of 1763 mass features in the positive mode and 718 mass features in the negative mode. ANOVA identified metabolites that differed significantly among genotypes (*p* < 0.05) in the positive ion mode (*n* = 354) and in the negative ion mode (*n* = 162). A full list of features identified by ANOVA are presented as Supplementary Data, and Fig. 3 illustrates variation in a subset of these features among genotypes. U41 displayed greater variability in metabolite concentration among replicates compared to genotypes U25 and U44, but this variability did not appear to relate to donor colony (A versus B; Fig. 3). PCA components 1 and 2 described 43.6% (positive ion mode) and 44.6% (negative ion mode) of the total variance among genotypes. In PCA models derived from both the positive and negative ion mode, U44 clustered separately from both U25 and U41, however U41 had greater within-group variability compared to the other two genotypes, resulting in overlap with U25 (Fig. 4). A PLS-DA model was not well-validated, and was therefore not used to assess data derived from LC-MS. Features were identified by searching against an internal retention time metabolite library of 1100 compounds as well as the Kyoto Encyclopedia of Genes and Genomes database (https://www.genome.jp/kegg). A full list of compounds resolved by LC-MS, primarily consisting of level 1 (confirmed structure), level 3 (tentative candidates), and level 5 identifications (exact mass, *m/z*)^35^, is provided as Supplementary Data.

*Mummichog* pathway enrichment analysis^36^ was used to identify the metabolic pathways that differed in activity among genotypes. *Mummichog* software finds all possible metabolite matches corresponding to *m/z* features resolved by LC-MS and compares these against a reference metabolic network in order to compute the most probable pathways driving separation among groups^36^. Aminoacyl-tRNA biosynthesis was identified as a pathway that varied substantially among genotypes using LC-MS data from both the positive and negative mode. Lysine biosynthesis (positive mode), as well as phenylalanine, tyrosine, and tryptophan biosynthesis (negative mode) differed based on genotype. In addition, metabolism of the amino acids arginine, proline, cysteine and methionine (positive mode) as well as glycine, serine, and threonine (negative mode) varied among genotypes. Purine (positive mode) and pyrimidine (negative mode) metabolism were also identified as important metabolic pathways driving separation among genotypes. A full list of metabolic pathways identified by *Mummichog* is provided as Supplementary Data.

## Discussion

As threats to coral reefs grow, interest in better understanding intraspecific differences in coral survival and performance is increasing. A number of studies have documented genotype-specific differences in growth, stress tolerance, and/or survival^9,25,26,30,37,38^. Metabolomic profiling has the potential to provide insight into the physiological drivers of these intraspecific differences. As a first step toward this goal, we tested whether metabolomic profiles vary among unique genotypes of the staghorn coral *A*. *cervicornis* in a common garden. Using both ^1^H-NMR and LC-MS approaches, we show that metabolomic profiles indeed varied among the genotypes sampled. However, within-group variability in the metabolome for genotype U41 contributed to varying degrees of overlap with metabolomic profiles for the other two genotypes in PCA models. Although within-genotype variability could complicate intraspecific metabolomic profiling, this study demonstrates that distinct metabolomic profiles can be resolved for some genotypes of the same coral species in a common garden setting.

Interestingly, the genotypes compared in the present study were previously found to have unique growth and stress tolerance phenotypes^25^. Links between phenotype and metabolomic profile have been found in previous studies of plant systems^39,40^. For some agricultural species, metabolite biomarkers associated with desirable traits have been discerned^41,42^. Our findings of distinct metabolite profiles among unique genotypes support the idea that future studies could identify metabolite biomarkers for key *A*. *cervicornis* traits, including, but not limited to, disease resistance^10^ and thermotolerance^9,25,30^. This concept is also supported by a previous study of the soft coral *Sinularia*, which documented distinct sesquiterpene signatures among species with different morphological and anatomical traits, indicating a possible link between phenotype and metabolome in corals^43^. At present, identification of coral genotypes that possess traits of interest can involve painstaking repeated measurements *in situ*^25,44^ or long-term manipulative experiments^10,45^, which are time-consuming and costly. Recent attempts to identify transcriptomic biomarkers for thermotolerance and other traits of interest in *A*. *cervicornis* were complicated by high within-genotype variability in gene expression^46^. The present study suggests that metabolite biomarkers could be easier to distinguish for some *A*. *cervicornis* genotypes, and this could therefore be a productive area for future study.

While host genotype is a key factor in determining metabolite profile, the coral holobiont metabolome ultimately reflects the physiological activity of multiple symbiotic partners. These partners include the coral host, *in hospite* Symbiodiniaceae, and the coral-associated microbial community^18^. Our study was limited to a comparison of holobiont samples with host genotype as a factor. Future analyses should consider incorporating data on host genotype, Symbiodiniaceae strain, and microbiome community structure in order to gain a fuller understanding of the physiological role of each partner and their individual contributions to the metabolome. Although *A*. *cervicornis* is known to associate almost exclusively with the dinoflagellate taxon *Symbiodinium ‘fitti’* nomen nudum (ITS-2 type A3)^38,46–49^, this taxon encompasses a number of genotypes, or strains, that can have different physiological characteristics^19^. The complex interplay of host genotype and symbiont strain ultimately drives a number of phenotypes in corals^19,50^, and it is therefore likely that both partners influence the holobiont metabolome. Similarly, the microbiome is increasingly understood as a key factor affecting host physiology, and is also known to drive differences in the coral metabolome^21^. Incorporating data on Symbiodiniaceae identity and microbial community structure in future coral metabolomics studies could improve understanding of the role of these symbionts in coral physiology, and help clarify the scope of each partner’s contribution to the holobiont metabolome.

Although the identity of the coral host and symbiotic partners are highly important in driving physiological differences among corals, the environment also plays a key role. The present study was conducted in a common garden to control for the effect of environment in order to isolate differences among genotypes. Coral nurseries can be valuable tools for such studies^25,26,31^. Additionally, a number of studies have examined genotype-environment interactions^51,52^ during outplanting and found considerable phenotypic plasticity across varying sites^9,53^. Although little information is available regarding genotype-environment effects on the coral metabolome, environmental conditions are known to play a key role in determining metabolomic profile in other organisms^54–56^. Future studies of corals should consider plasticity in metabolite profiles among diverse sites. Because corals are sessile organisms, they could be useful for investigating metabolomic shifts following transplantation to differing sites from a common garden. Such information could provide insight into site selection for restoration activities.

In addition to spatial variation, temporal changes in abiotic conditions within a site can occur and potentially affect the metabolome. Temperature data suggest low thermal stress in 2016 compared to 2015. Daily mean temperature exceeded 31 °C for two 10-day periods in late summer 2015, inducing sub-lethal bleaching^25^, but daily mean temperature did not exceed this threshold in 2016. Because samples in this study were not collected during a period of thermal stress, key metabolites involved in physiological responses to elevated temperature may not be present in metabolomic profiles reported here. Recent studies have demonstrated metabolomic shifts in cnidarians, including corals, in response to thermal stress^20,24,57^ and combined thermal and chemical stress^23^. Thus, differences in metabolomes among genotypes could be more or less apparent depending on abiotic conditions at the time of sampling. To better capture the full range of metabolites associated with a particular genotype, time series designs could be incorporated into future studies. In particular, sampling points during periods of high and low thermal stress could be useful in identifying metabolites associated with thermotolerance.

Although we were able to resolve distinct differences in metabolite profiles for all three genotypes when ^1^H-NMR data were assessed using PLS-DA, higher within-genotype variability in metabolite profile was found for genotype U41. When metabolomic profiles were examined using PCA, this variability resulted in overlap of profiles between U41 and one or both of the other two genotypes based on LC-MS and ^1^H-NMR, respectively. ANOVA also indicated varying concentrations of key metabolites among U41 replicates, with no apparent pattern based on the specific colony sampled. Together, these results may indicate intracolonial variability in the metabolome for genotype U41. Interestingly, U41 was found to have within-genotype differences in linear extension in our previous study^25^. Within-colony differences in metabolic processes related to linear extension could explain variability in the metabolome for genotype U41. Together, observations of within-genotype differences in growth and intracolonial variability in metabolomic profile for genotype U41 could indicate intracolonial variation in genotype^58^. Potential intracolonial variation in genotype in *A*. *cervicornis*, and particularly in genotype U41, warrants further study.

In addition to possible intracolonial variation in genotype, our study was also complicated by a lack of metabolite databases for *A*. *cervicornis* and other coral species^13^. Regardless of this challenge, we were able to identify a number of putative compounds that varied among genotypes. Few significantly different putative metabolites were identified using both ^1^H-NMR and LC-MS approaches; these include threonine, homoserine, leucine, creatine, and isoleucine (see Supplementary Data). The differences in the number and identity of putative metabolites resolved by ^1^H-NMR and LC-MS may highlight the varying strengths of each method. For example, LC-MS has higher sensitivity compared to ^1^H-NMR, and can therefore detect a higher number of mass features, including very rare compounds^33^. However, the types of features detected may be dependent on the chromatography technique applied. ^1^H-NMR captures a smaller number of metabolites, but is less susceptible to matrix effects, produces quantitative estimates of concentration without standards curves, and is therefore highly reproducible across labs, making it a valuable tool for untargeted metabolomics^33^. Application of both methods in the present study therefore provides a more comprehensive metabolomic profile of *A*. *cervicornis* compared to the use of either technique alone.

^1^H-NMR identified 15 chemical shifts highly important in driving separation among the three genotypes compared in this study (VIP > 2; Table 1). These chemical shifts are primarily classified as carbohydrates and aliphatic compounds, however our solvent likely extracted more polar entities compared to non-polar compounds, such as fatty acids. Regardless of this limitation, ^1^H-NMR was able to putatively identify trimethylamine N-oxide (TMAO) as the compound with the most variability among genotypes. In many marine organisms, TMAO is well-known as an important osmolyte that prevents the damaging effects of urea buildup^59,60^ and hydrostatic pressure^61^ on proteins. The function of TMAO in the coral holobiont is less clear, however synthesis of this compound has been linked to protection against hydrostatic protein damage in other cnidarians^62^. Metabolomic studies of other acroporids were also consistent with our putative identification of choline and malonate^63^ as well as homoserine^20^ in *A*. *cervicornis* in the present study. Unfortunately, there is currently a lack of published information to link these or any other highly significant metabolites in our study with potential physiological roles in the *A*. *cervicornis* holobiont.

A number of important mass features driving separation of profiles for the three genotypes examined were also resolved by LC-MS. In contrast to ^1^H-NMR results, very few carbohydrates were resolved by LC-MS. Carbohydrates are known to fragment readily using MS-based techniques, and can therefore be more difficult to resolve^64^. Despite these challenges, a number of metabolites were identified or putatively identified based on LC-MS data from the positive and negative modes (see Supplementary Data). One such metabolite was catechin, a well-studied flavonoid known for its antioxidant properties^65^. Interestingly, a recent study found that addition of catechin in the laboratory could prevent bleaching in thermally-stressed *Porites astreoides*^66^. Targeted metabolomics studies could confirm the presence of catechin in *A*. *cervicornis*, and also strive to better understand its potential role in coral thermotolerance. A number of other organic compounds common in many metabolic processes also varied among genotypes. Additional studies could reveal more about specific roles of these metabolites in coral physiology and shed light on why they vary among genotypes. Interestingly, compounds associated with human activity, such as coronene and flusilazole were also identified by LC-MS. Although corals are known to absorb pollutants from the marine environment^67–69^, it is unclear why absorption of such compounds would vary among genotypes in a common garden. It is also possible these compounds were misidentified during LC-MS analysis or unintentionally introduced during sampling. This question could be resolved in the future by performing MS/MS to identify putative metabolites of interest with a higher level of certainty compared to the present study.

A number of metabolic pathways were found to drive separation of metabolite profiles among genotypes in both the positive and negative mode for LC-MS. Both datasets identified biosynthesis of aminoacyl-tRNA, a critical component of translation and protein synthesis^70^, as the most important pathway driving separation. Pathways for the synthesis and metabolism of a variety of amino acids as well as purine and pyrimidine metabolism varied based on genotype, potentially also suggesting differences in transcriptional and translational activity among the three coral genotypes. Future transcriptomic and proteomic analyses of these genotypes could aid in identifying specific gene activity and proteins driving separation among genotypes, as well as their physiological role within the holobiont.

Metabolomics is an emerging technology in coral reef science, and clearly linking individual metabolites to physiological processes in corals is an important next step in this field. Better understanding of metabolomic variation in corals can assist with future efforts to identify key physiological processes related to growth and stress tolerance, and potentially support selection of robust genotypes for restoration and other interventions^2,3^. Future research on the metabolome of *A*. *cervicornis* and other coral species can aid in building databases to improve metabolite identification. Additional work and improvements in coral bioinformatics are needed to begin linking specific metabolites to the physiological processes and identifying possible biomarkers for traits of interest.

## Methods

### Sample Collection and Extraction

Corals used in this study were collected from an established coral nursery operated by the Coral Restoration Foundation (CRF) and located four miles offshore of Tavernier, FL. All *Acropora cervicornis* genotypes in this nursery were previously determined to be unique via microsatellite genotyping performed by the Baums lab at Penn State University (unpublished data). Associated Symbiodiniaceae were not genotyped during this study.

In December 2016, two colonies from each of three genotypes (U25, U41, and U44) were removed from grow-out structures at a depth of approximately 8 m in the nursery and brought to the surface intact. Corals remained submerged during sample collection to minimize stress. Diagonal pliers were used to clip ~3 cm actively growing branch tips on each colony. A total of five replicate holobiont samples were collected from genotypes U25 and U44, with three tips collected from the first replicate colony (A) and two collected from the second replicate colony (B). Six replicate tips were collected from genotype U41, with four tips collected from the first replicate colony (A) and two collected from the second replicate colony (B). Tips were placed in 20 mL scintillation vials containing 10 mL of 100% methanol <5 seconds following removal from each colony, and vials were immediately placed on ice in a cooler. Immersion in methanol has been shown to be an effective method for quenching metabolic activity^71,72^, and was more conducive to offshore field collections compared to snap freezing in liquid nitrogen. Following sample collection, colonies were returned to the nursery and an existing logger (HOBO Pendant® UA-002-64, Onset Corporation) was downloaded to determine temperature from July–December 2016, inclusive of the sample collection period. Samples were transported to shore and stored at −20 °C overnight. Samples were transported back to the laboratory on ice and were again stored at −20 °C overnight.

The next day, vials containing intact holobiont fragments in methanol were placed in a single rack and shaken for 5 minutes, then individually vortexed for 30 seconds per sample. Holobiont samples were allowed to settle for one hour at −20 °C. One mL of extract from each sample was transferred to clean 1.5 mL microcentrifuge tubes and centrifuged at 20,000 g for 5 minutes. The supernatant was then transferred to a new 1.5 mL microcentrifuge tube and stored at −80 °C until processing.

### ^1^H-NMR Profiling

All metabolomic analyses were performed at the Southeast Center for Integrated Metabolomics (SECIM) at the University of Florida. Coral holobiont extract (in methanol) was added to double distilled water (1:2 v/v of sample to water), then flash freeze lyophilized (Labconco) until dry. Lyophilized dry powder was re-suspended in phosphate buffer in deuterium oxide (D_2_O) at pH 7. The final volume for the ^1^H-NMR samples was 60 μL (in a 1.5 mm tube) with 90% (v/v) of deuterated 50 mM sodium phosphate buffer (pH 7) with 2 mM of ethylene diamine tetra-acetic acid (EDTA). The remaining 10% (v/v) was occupied by an internal standard [5 mM D_6_−4,4-dimethyl-4-silapentane-1-sulfonic acid (DSS-D_6_) and 0.2% sodium azide (NaN_3_) in D_2_O; Chenomx, Inc.].

All ^1^H-NMR spectra were collected with a 14.1 T NMR system, equipped with a CP TXI CryoProbe and Avance II Console (Bruker Biospin). The first slice of a NOESY pulse sequence (tnnoesy)^73^ was used to acquire proton spectra consisting of 1 s relaxation delay (d1), 64 scans (nt), 100 ms mixing time, with 4 s acquisition time over a spectral window (sw) of 7211.54 Hz. Samples were acquired at room temperature (25 °C). Before Fourier transformation, acquired spectra were further processed with a line-broadening factor of 0.5 Hz and zero filling to 65,536 points. MestReNova 11.0.0-17609 (Mestrelab Research S.L.) was utilized to process the spectra. Identification and quantification of the metabolites from the ^1^H-NMR spectra was done using Chenomx NMR Suite 8.2 (Chenomx, Inc.).

### LC-MS Global Metabolomics

LC-MS global metabolomics samples were prepared by protein precipitation. Briefly, 5 µL of internal standard mixture prepared in-house consisting of labeled amino acids were spiked into each 25 µL sample. Extraction was done by adding 200 µL of 8:1:1 Acetonitrile:Methanol:Acetone to the sample. Samples were held at 2–8 °C for 30 min to allow protein precipitation. Samples were centrifuged at 20,000 × g for 10 minutes at 4 °C. From each sample, 190 µL supernatant were collected and dried completely under nitrogen at 30 °C. Samples were reconstituted with 25 µL of reconstitution solution containing injection standards. Samples were mixed thoroughly, held at 2–8 °C for 10 min, and centrifuged at 20,000 × g for 10 min at 4 °C. Supernatants were transferred to vials for LC-MS analysis.

Global metabolomics profiling was performed on a Q Exactive Orbitrap mass spectrometer with UltiMate 3000 UHPLC (Thermo Fisher Scientific). All samples were analyzed in positive and negative heated electrospray ionization with a mass resolution of 35,000 at *m/z* 200 as separate injections. Separation was achieved on an ACE 18-PFP 100 × 2.1 mm, 2 µm column (MAC-MOD Analytical) with mobile phase A as 0.1% formic acid in water and mobile phase B as acetonitrile. This is a polar embedded stationary phase that provides comprehensive coverage, but does have some limitation in the coverage of very polar species. The flow rate was 350 µL/min with a column temperature of 25 °C. 4 µL were injected for negative ions and 2 µL for positive ions.

MSConvert (ProteoWizard 3.0) was used to convert raw files to open format. MZmine 2 was used to identify features, deisotope, align features, and perform gap filling to fill in any features that may have been missed in the first alignment algorithm. All adducts and complexes were identified and removed from the data set. The data were searched against SECIM’s internal retention time metabolite library of 1100 compounds and subsequently searched against KEGG for putative identification.

*Mummichog*, a program available on metaboanalyst.ca, was used to identify metabolic pathways driving separation among genotypes. The p-value and t.score along with the *m/z* value were used for pathway searching with a p-value cutoff of 0.01 and mass accuracy of 5 ppm in positive mode and 10 ppm in negative mode. A metabolic pathway reference library for the model species *Arabidopsis thaliana* was used in this analysis, as no coral libraries were available.

### Statistical analysis

All statistical tests were conducted at a significance level of α = 0.05. ^1^H-NMR spectra were pre-processed in MestReNova 11.0.0-17609 (Mestrelab Research S.L.) before extracting data for the Metaboanalyst analysis. Fourier transformation, calibration with respect to an internal standard (DSS) peak at zero ppm, phase correction, base-line correction (using the Spline method), removal of inconsistent lipid/proteins regions and the water peak, and local alignment of many peak regions (to adjust chemical shift variability issues due to pH or other technical/instrumental issues) were performed. Data binning of 0.04 ppm was performed to restrict the dimensionality of the data, with removal of the downfield >9.5 and upfield <0.5 regions not containing any metabolic data. Processed data were analyzed in Metaboanalyst^74^. Missing value estimation was performed by removing features with greater than 50% missing values then replacing these features with half of the minimum positive values from the original data. Data filtering was performed with interquartile range (IQR) to remove peaks that were unlikely to be of use in data modeling. Probability quotient normalization was performed to reduce any possible variation in total signal intensity between the groups^75^, including possible bias that might have arisen because of sample handling and potential variability in the total amount of tissue per sample. Pareto scaling (mean-centered and divided by the square root of the standard deviation of each variable) was used to provide equivalent weight among the variables.

For ^1^H-NMR data, abundance of metabolites among three different genotypes of corals was analyzed via multivariate statistical analysis. The analysis used 15 spectra for a set (five samples per genotype). For LC-MS data, any metabolites present in <80% of samples were discarded. If data met that criterion, any missing values were imputed with half the minimum value of the reported data. Next, the data were filtered for relative standard deviation to remove metabolites with a high degree of variability. Finally, the data were normalized to the total ion signal, log transformed and autoscaled (mean-centered and divided by the standard deviation of each variable).

The web-based metabolomics data processing tool MetaboAnalyst 3.0^74^ was utilized to perform one-way analysis of variance (ANOVA), principal component analysis (PCA), and partial least square discriminant analysis (PLS-DA) for both LC-MS and ^1^H-NMR data. All *p*-values were FDR corrected and only values of 0.05 or less were reported. The data met all assumptions of ANOVA. ANOVA models based on either ^1^H-NMR or LC-MS data used concentration of a given compound (i.e. integrated peak height, normalized to the total ion signal) as the response variable, with genotype as a factor. Significance of PLS-DA models was assessed with permutation tests (consisting of 1000 permutations) and leave one out cross-validation (LOOCV). Robustness of PLS-DA models was validated by calculating Q^2^. For LC-MS, data from positive and negative ion modes were analyzed separately. Variable Importance in Projection (VIP) was used to summarize the importance of each variable (i.e. metabolite) in driving separation among treatments (i.e. genotypes) in the PLS-DA models^76,77^. Compounds with a VIP values >1 are generally considered to be influential in PLS-DA models^76,78^. The present study used the conservative cutoff value of >2 to identify highly important compounds driving separation.

## Supplementary information

Dataset 1

## Data Availability

All data generated or analyzed during this study are publicly available via Metabolomics Workbench (Project ID: PR000747) and as Supplementary Data.
